# Creativity as emulation: the cultural basis of creative cognition

**DOI:** 10.3389/fpsyg.2024.1364596

**Published:** 2024-04-08

**Authors:** Steven Brown

**Affiliations:** Department of Psychology, Neuroscience, & Behaviour, McMaster University, Hamilton, ON, Canada

**Keywords:** creativity, divergent thinking, emulation, analytic thinking, cultural evolution

## The standard view of creativity in psychology

**Figure 1:**
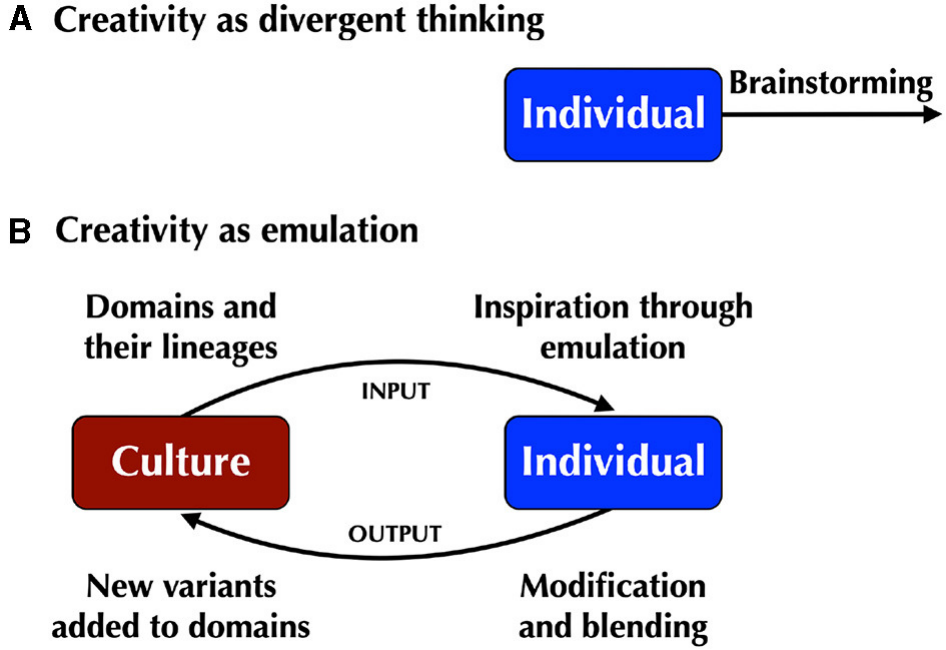
Culture is both the input to and the output of the creative process. **(A)** Divergent thinking is the standard model of the creative process in the psychology literature, but it lacks any concept of culture or society. **(B)** In a systems model of creativity, culture is both the input to and the output of the creative process. Creators operate within domains having historical lineages of products that serve as major sources of inspiration for these people. Much creation occurs through a process of emulation of these products by means of modification and blending. This includes self-emulation of one's previous work. The outputs of creators become new variants in the cultural evolution of these domains, adding new products to their stylistic lineages.

In this article, I attempt to provide an alternative to the standard model of creative cognition in the psychological study of creativity by arguing that creativity is a process of end-product emulation. Since the 1960's, the psychology of creativity has honed in on a mechanism that is, at once, descriptive and prescriptive, namely Guilford's (1967) concept of divergent thinking. This process is not only assumed to be an analytical account of how creative cognition operates in the minds of creators, but a prescription for how a person should enhance their level of creativity: engage in divergent brainstorming (Figure 1A). The psychological study of creativity has used divergent-thinking tasks as *diagnostic tools* to assess who is more vs. less creative in the general population in order to predict their future success as creators (Guilford, 1950; Plucker et al., 2019). The most popular of these laboratory tests is the Alternate Uses task (Guilford, 1967; Torrance, 1974, 1988), which requires participants to come up with as many unusual uses for a common object as possible in a 2-min time period. These uses are then rated by judges (i.e., the researchers) for their fluency, flexibility, originality, and elaboration (Runco, 2010). The end result is a simple quantitative measurement of trait-level creativity that can be tested against other types of data, like neuroimaging findings (both functional and structural) and genetic data (e.g., Zhang et al., 2014; Beaty et al., 2016). Divergent thinking and its diagnostic tools have received widespread adoption in the psychology of creativity (Benedek et al., 2019).

## The systems model of creativity

There are significant problems with this perspective (Baer, 2016; Sternberg, 2020; Brown and Kim, 2021; Brown, 2022). The principal one is that it eliminates any connection with culture or society, and instead places an exclusive focus on the isolated individual as well as on single-shot production tasks that have no past or future. Csikszentmihalyi (1988) has provided the most substantial alternative to the divergent-thinking perspective, called the “systems model” of creativity. In this model, *culture is both the input to the creative process and its final output*, as shown in Figure 1B. Virtually all creators work within a domain. Creative work is highly institutionalized and is driven by the constraints of a domain. Domains provide an historical lineage of traditional products that the creator inherits and is educated on. At the output end of the model, the domain is the receiving place for the creator's own products. Creators are the major source of new variants in the cultural evolution of a domain (Csikszentmihalyi, 1988; Fogarty et al., 2015; Gabora, 2019), thereby increasing the diversity of products in that domain.

An aspiring symphony composer, for example, will not only have access to several centuries' worth of orchestral music – in both notated and recorded form – but the person will hope that any composed symphony will be performed by an established orchestra during a concert season, and that it will be appreciated by the audience. The aesthetic appraisal of consumers functions as a strong force of cultural selection in an evolutionary model of culture (Brown, 2022). Gatekeepers act as a critical intermediate in this process (Csikszentmihalyi, 1988). They serve as a selective force in determining which products the people of a domain will get exposed it. Examples of this include the reviewers, action editor, and publisher of the present article. Gatekeepers have a significant effect on the adoptability – and thus transmissibility – of creative products (Rogers, 2003).

## Creativity as emulation

Gilfillan (1935) argued that innovative products have “abundant and clear causes in prior scientific and technological development” [quoted in Rhodes (1961), p. 309]. Weisberg (2020) formalized this idea into the concept of “analytic thinking,” arguing that creative ideas are based on antecedent ideas and products, an associative process that he calls “thinking inside the box.” As he writes: “creative thinking is based on *continuity with the past*. It begins with old ideas and attempts to extend them to new situations. Creative thinking does not make a radical break with the past; we build on the old to get to the new” (p. 14, emphasis in the original). Continuity implies a strong link between creativity and both problem solving and problem finding (Csikszentmihalyi, 1988). Although Weisberg's model is not grounded in cultural evolution, his notion of continuity in domains has strong similarities with the concepts of lineages, genealogies, and phylogenies in analyses of the evolutionary patterns of culture (Mace and Pagel, 1994; Tehrani, 2011, 2013), where enduring creative changes establish new traditions in a domain.

I would like to invoke a similar idea to Weisberg's concept of analytic thinking in considering the psychological basis of creativity, namely the notion of emulation. In cognitive psychology, emulation is distinguished from imitation in that, while imitation is about copying the *process* of performing an action, emulation is about copying the *end-product* of that action (Tomasello et al., 1993; Heyes, 2021). A composer can emulate Tchaikovsky's music by creating music in his style without replicating the specific process that Tchaikovsky went through in composing his music. Emulation only considers the product, not the process, of creativity. Emulation also has a colloquial meaning outside of the cognitive literature to signify modeling oneself after another person. For example, we might say that a daughter emulates her mother by becoming a doctor.

I would argue that creators, instead of sitting around brainstorming divergently, emulate the products of other creators in their domain, as well as their own previous work (Brown, 2022). These products are the major sources of inspiration for creators. Emulation is a central part of the pedagogical process in becoming a creator in a domain. People use their teachers as role models, and begin their professional careers as emulators of their ideas and products. Kleon's (2012) book *Steal Like an Artist* presents a popular account of the emulation model of creativity in which creators are advised to strategically select inspirational role models and to emulate their products as much as possible as a means of discovering their own uniqueness as creators. “Steal like an artist” is a very different prescription for aspiring creators than “think divergently.” However, I suspect that it is a far more accurate description of what creators actually do in their professional work.

**Figure 2:**
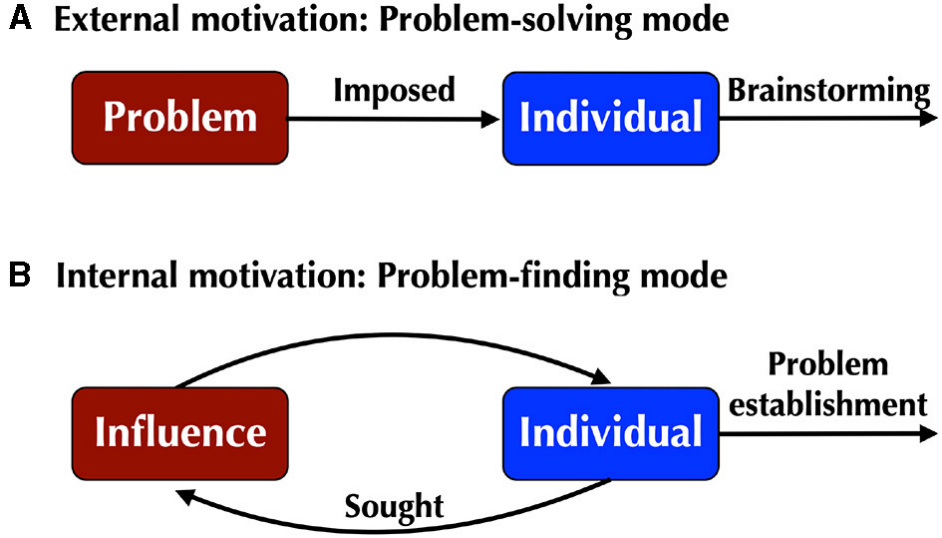
Different strategies for creation. **(A)** When creative work is externally motivated by an imposed problem, it tends to proceed through a problem-solving mode that incorporates brainstorming processes. **(B)** However, when creation is internally-motivated, it is more likely to proceed via a problem-finding mode that is associated with emulation, including self-emulation and voluntary exposure to the ideas of others.

The strategies of creativity are most likely influenced by the driving forces for creation (Figure 2), as seen in “typological” approaches to creativity (Kozbelt et al., 2010). When the impetus for creative work is external to oneself as an imposed problem – such as when a boss requests that a subordinate create a new ad campaign for their company, or when a global pandemic suddenly emerges – then brainstorming is undoubtedly a necessary first step in the process of solving a new and unexpected problem. However, when creative work is internally motivated, then the emulative approach probably predominates, as evidenced by the stylistic continuity of the output of many professional creators across their careers (Galenson, 2009). This strategy is more of a problem-finding mode in which the creator often seeks to extend the trajectory that was established in their previous work. Even when stylistic breaks do occur in the lineage of a creator's output, it cannot be assumed that such discontinuities arose through divergent brainstorming. For example, Arnold Schoenberg's formulation of the revolutionary compositional technique of serialism (or 12-tone music) in the 1920's came about as a response to many years of frustration over how atonal music could be produced as large-scale works, rather than the miniature compositions that dominated the early years of atonal music (Rosen, 1975). Serialism was an extension of Schoenberg's work on atonality over the preceding 15 years. So, the psychological issue of how creators stumble upon their revolutionary ideas is an historical question, not something that can be captured in a few minutes through a psychometric test.

## The mechanisms of creativity

What are the mechanisms of creativity from an emulative/analytic perspective? Boden (2010) argues that, when all is said and done, there are two principal mechanisms of creativity when seen from the standpoint of products, what I will refer to as *modification* and *blending*. The vast majority of creation is probably little more than an emulative modification of some existing product or product-type. This can be thought of most intuitively as the generation of a small improvement in a product. Sternberg (1999) refers to this process as forward incrementation, since the improvement is generally an incremental change compared to what came before. We can think of this mechanism as the underlying process of “cumulative culture” in human evolution (Tomasello et al., 1993; Tennie et al., 2009), in which products evolve over time through the gradual accumulation of functional improvements. Cumulative culture is considered as a defining feature of human evolution (Mesoudi, 2017). Emulation might provide a psychological basis for it. Creativity is not merely a *component* of cultural evolution, but a catalyst for the emergence culture to begin with (Donald, 1991; Fogarty et al., 2015; Gabora, 2018). Hence, it makes sense to think of a co-evolutionary relationship between creativity and culture.

The other major mechanism of creativity aside from modification involves a conceptual blending of two or more ideas/products, what we can think of as recombination, reframing, association, or comparison (Koestler, 1964; Finke et al., 1992). Boden (2010) refers to this process as combinatorial creativity, and defines it as “making unfamiliar connections between familiar ideas” (p. 42), resulting in novel fusions or hybrids. Blending is still based on emulation, but it uses two sources as its raw material (instead of one), and seeks novel means of combining them. This includes the use of metaphors (Gruber and Davis, 1988) and analogies (Weisberg, 2020). It also includes the reframing and restructuring of existing ideas. Meyer (1989) provides a brilliant account of innovation in the history of Western classical music. He contends that “the most common and important source of strategic innovation is manipulation – ordering or modifying already existing stylistic means in new ways” (p. 123). Mechanisms for doing this include permutation, combination, displacement, and extrapolation.

Thinking about creativity from the standpoint of emulation highlights the important point that existing products are both an inspiration for the creative process and a major constraint on it. Emulation is both a blessing and a curse for creativity. This is mentioned by Sternberg and Lubart (1995) as the need of creators to know enough about a domain, but to not become entrenched in its stylistic features. Existing products function as psychological *attractors* that limit people's imagination and that inhibit them from seeing beyond the products' features. This type of expertise trap is referred to as functional fixedness in the realm of problem solving (Duncker, 1945). Fixedness is an inescapable outcome of creativity being an emulative process. People find inspiration in the products of their domain, but these products also constrain their imagination, ensuring that most creativity is incremental in its degree of change compared to existing products.

## Creativity as style change

I argued in Brown (2022) that that we can fruitfully use the concept of style to think about how creativity operates in a systems model of creativity, both historically and geographically (see also Meyer, 1989; Chan, 2015). Creativity is fundamentally a mechanism of stylistic change and product-level diversification within the lineages of domains, leading to branchings in cultural phylogenies. Most of this change occurs in an incremental manner (Sternberg, 1999; Weisberg, 2020). Style analysis allows us to see the historically contingent nature of creative change (Boas, 1927; Csikszentmihalyi, 1988; Weisberg, 2020), and this highlights the critical role that emulation plays in the creative process. Importantly, style-based concepts apply both to a culture overall – i.e., historical changes to the domains of a culture – and to individual creators, namely stylistic changes across a particular creator's career trajectory (Simonton, 1980; Meyer, 1989; Galenson, 2009).

Every domain has certain stylistic boundaries that define what people in that domain consider to be acceptable products. Most new products sit comfortably within these domain-defining boundaries, in keeping with Weisberg (2020) concept of “thinking inside the box.” However, there are occasionally revolutionary works that push the stylistic boundaries of a domain to uncomfortable levels. When Igor Stravinsky's ballet *The Rite of Spring* premiered in Paris in 1913, the score's atonality and arrhythmicity made people think that it was not a legitimate example of music. It had pushed the stylistic boundaries too far from what existed previously in classical music. It is important to point out that most revolutionary products, despite being highly novel and creative, never make it past gatekeepers and become adopted by members of a domain.

## Conclusions

The divergent-thinking account of the creative process through brainstorming looks at creativity in a social vacuum. Creativity should instead be thought of as one component in a cultural evolutionary model that connects creators reciprocally with both domains and consumers, as shown in Figure 1. Creators are inheritors of lineages of creative products in their domain, and they contribute new variants to these evolving lineages. Most creativity is based on an associative process of analytic thinking and emulation that achieves a modification of existing products or a blending of products from two domains. As a result, most creative products are incremental changes compared to what came before, sitting comfortably within the stylistic boundaries of a domain, often conforming with a creator's previous work in that domain. This gradualism underlies the cumulative nature of cultural change that serves as a hallmark feature of human cultural evolution.

What is desperately needed is to move beyond the 2-min diagnostic tests of divergent thinking in the laboratory and instead develop a large-scale ethnographic research program into the practices that creators employ “*in vivo*” in their daily work, looking across many domains (e.g., Glaveanu et al., 2013; Ness and Dysthe, 2020). If it turns out that the majority of creators engage in divergent brainstorming as the critical means for developing their ideas and optimizing the novelty of these ideas, then the perspective of this article will be shown to have limited applicability. However, if it is shown that most creators take inspiration from the products that they are exposed to in their domain and then fashion changes to these products, or create hybrids or reframings of them, then it will provide an important grounds for seeing creativity as a process of end-product emulation and analytic thinking. This cannot be decided in the laboratory. Ethnographic and historical analyses are the only means of addressing this issue (Ghiselin, 1985; Weisberg, 2004; Arnheim, 2006; Simonton, 2010; Doyle, 2022). This is all the more important in fields of collaborative creativity (John-Steiner, 2000; Sawyer and DeZutter, 2009), where the creative process is negotiated by a group of creators, rather than carried out by a lone individual.

## Author contributions

SB: Conceptualization, Writing – original draft.
